# Qualitative research concerning physiotherapy approaches to encourage physical activity in older adults with dementia

**DOI:** 10.1371/journal.pone.0289290

**Published:** 2023-07-27

**Authors:** Masami Yokogawa, Yoshimi Taniguchi, Yumi Yoneda

**Affiliations:** 1 Department of Physical Therapy, Faculty of Health Sciences, Institute of Medical, Pharmaceutical and Health Sciences, Kanazawa University, Kanazawa, Japan; 2 Department of Clinical Nursing, Faculty of Health Sciences, Institute of Medical, Pharmaceutical and Health Sciences, Kanazawa University, Kanazawa, Japan; 3 Rehabilitation Division, Enyama Kenko Clinic, Nanao, Japan; Faculty of Associated Medical Sciences, Chiang Mai University, THAILAND

## Abstract

**Background:**

Physical exercise is known to improve the level of activities of daily living and physical function in people with dementia; however, symptoms of dementia often pose challenges when implementing physical therapy. This study aimed to elucidate how physiotherapists (PTs) engage with older adults with dementia to encourage exercise and participation in physical activity.

**Methods:**

In this qualitative study, four PTs working with older adults with dementia in long-term care facilities in Japan were recruited and interviewed. We used a modified grounded theory approach to assess how PTs engaged with older adults with dementia during physiotherapy sessions.

**Results:**

Based on PT responses, five categories of engagement were identified: “make structured preparations for clients to begin physical activity,” “link exercise therapy to a client’s daily life,” “discover changes in daily life,” “ascertain cognitive function,” and “accommodate client differences.” Concepts were derived under each category. The category “make structured preparations for clients to begin physical activity” served as a preceding stage for PTs to engage with older adults with dementia. PTs linked exercise therapy to each client’s daily life activities to encourage voluntary participation in daily physical activity. PTs ensured the performance of routine patterns of movement and modified these movement patterns per clients’ differing paces.

**Conclusion:**

PTs provided exercise and movement training based on various degrees of client involvement and made structured preparations for clients to begin physical activity that were linked to exercise therapy. Our findings may prompt PTs to encourage older people with dementia to participate in physical therapy and benefit from exercise.

## Introduction

Dementia is a syndrome involving more severe cognitive decline than the degree expected from the usual consequences of biological aging [1]. The number of people with dementia worldwide is estimated to increase from 57 million in 2019 to 152.8 million in 2050 [2]. The same report estimated that Japan, compared with other countries, has the lowest rate of increase in patients with dementia. However, the affected population is expected to increase from 4.12 million to approximately 5.24 million people. Since the incidence of dementia is known to increase exponentially with age [3], and population aging is a global phenomenon, older people in healthcare should be approached with the assumption of having dementia.

Physiotherapists (PTs) working in rehabilitation provide services to maintain and improve people’s movements and functions [4]. The opportunities for PTs to treat and care for older adults with dementia have increased in relation to long-term care, acute phase care, convalescent phase care, and in-home medical care. Older adults with dementia have been reported to have a lower physical function at discharge than those without [5], despite being a population more in need of physiotherapy. Nonetheless, previous studies reported that older patients with dementia who had sustained a hip fracture received less physiotherapy than those without dementia [6, 7]. Patients with dementia may be perceived as unsuitable candidates for rehabilitation therapy when introducing home care services owing to difficulties recalling or following instructions [5, 7]. In addition, some healthcare experts consider it challenging for older people with dementia to continually participate in rehabilitation programs due to the effects of core, behavioral, and psychological symptoms [8]. In recent years, exercise has been shown to improve the level of activities of daily living (ADL) and physical function [9–11] and reduce behavioral and psychological symptoms [11] in patients with dementia. Receiving less physiotherapy and failing to continually participate in it are challenges in benefiting from exercise; a solution to this might be for PTs to provide tailored exercise therapies specific to older adults with dementia.

Older adults with dementia may be clinically complex, and a greater level of dementia education is needed to address this complexity [12]. In this respect, identifying the practices and perceptions of PTs who engage with older adults with dementia could be helpful. Several studies have investigated PTs’ experiences and reflections when treating older adults with dementia [13–15]. Fjellman-Wiklund et al. reported on exercise in relation to older adults with dementia and listed factors essential for its efficacy, stating that knowledge gained through reflection-on-action and in-action is important [13]. Hall et al. described physiotherapy challenges facing older adults with dementia and the need to specifically modify and tailor physical therapy sessions to better meet the needs of this patient cohort [14]. PTs’ positive experiences in relation to rehabilitation possibilities for older adults with dementia have also been reported [14]. In an earlier study, we investigated how PTs working in long-term care became involved with older adults with dementia in providing physiotherapy services [15]. However, while PTs’ experiences with older adults with dementia have been reported [13–15], the process through which PTs provide treatment using appropriate interactions has not been fully elucidated. We hypothesized that determining this process could help older adults with dementia to participate in physiotherapy and benefit from exercise therapy. In this study, we aimed to elucidate how PTs engaged with older people with dementia to encourage exercise and participation in physical activity.

## Materials and methods

### Research design

In this qualitative study, PTs providing services to older adults with dementia were interviewed. A modified grounded theory approach (M-GTA) was used to analyze the interview responses. The M-GTA was used to understand and interpret details concerning the contextuality of the detail-rich data derived from semi-structured interviews wherein the interviewees were asked to speak as freely as possible [16, 17]. This study was undertaken in accordance with the Standards for Reporting Qualitative Research (SRQR) [18].

### Ethics

This study was conducted in accordance with the Declaration of Helsinki guidelines. All procedures were approved by the Medical Ethics Review Committee of Kanazawa University (Approval No.: 716). All PTs, clients, and clients’ family members, as well as the directors of the facilities where the PTs were affiliated, were provided with a verbal and written briefing on the purpose and details of this study prior to subsequently obtaining their written informed consent to participate.

### Research team

Two authors were PTs (MY, YY), one was a nurse (YT), and all were women. All members of the research team have experience with research in health science. YT has extensive experience in qualitative research about geriatric nursing and was involved in data analysis and supervision of this study.

### Participants and context

Snowball sampling [19] was used to recruit participants in a region of Japan where the authors resided. The region is located in the center of Honshu, Japan, and it combines both urban and rural elements. According to the White Book on Physical Therapy 2018 [20] edited by the Japan Physical Therapy Association, 3.3 members per 1,000 were over 65 years old, and 5.3% of the members were PTs in long-term care and day-care facilities for older people. The study region reflected the national average and PT situations in Japan. We contacted PTs working at adult day-care or long-term care facilities for older adults via email and telephone to ask whether their clients included older adults with dementia. By their referral, PTs experienced in dealing with older adults with dementia were invited to participate in the study, and four PTs who gave consent to participate were recruited. Inclusion criteria comprised PTs with >3 years of work experience in older adult facilities. Table 1 shows the attributes of the PTs and their clients; all PTs worked full-time. All involved facilities were privately operated.

**Table 1:** Characteristics of physiotherapists and their clients.

PT					Survey location	Clients					
No	Sex	Age	Number of years of experience	Number of years of experience in older adult facilities		Age	Sex	Outpatient/Admitted	BI	HDS-R	NM Scale
1	F	20	4	4	Daycare facility	84	F	Outpatient	95	15	39
2	M	50	25	19	Long-term care facility for older people	100	F	Admitted	0	0	11
						90	M	Admitted	10	0	13
3	F	40	23	18		81	F	Admitted	30	7	17
						91	F	Admitted	0	4	15
4	F	30	8	8		92	M	Admitted	65	7	25

BI, Barthel index; F, female; HDS-R, Hasegawa Dementia Scale-Revised; NM scale, N geriatric rating scale for mental states; M, male; PT, physiotherapist

Clients or their family members were invited to participate in this study via PTs. Six clients who gave consent to participate were asked for their cooperation to allow a researcher (MY) to observe the clients’ physiotherapy sessions. One client who visited the outpatient department could walk independently, while all other admitted clients required wheelchair assistance. The Barthel index was used to determine the ADL [21]. One client could perform almost all their ADL independently, whereas two clients required maximum assistance. The Hasegawa Dementia Scale-Revised (HDS-R), which is similar to the Mini-Mental State Examination (MMSE), is commonly used in Japan to assess overall cognitive function [22]. The test has high reliability with a Cronbach’s α coefficient of 0.90 and a confirmed correlation value of 0.94 with MMSE [22]. We collected data using the HDS-R concerning the clients’ cognitive function, as this tool is used for regular assessment at all facilities where the participants worked. The HDS-R has a maximum score of 30 points, and a score ≤20 points is considered suggestive of dementia [23, 24]. All clients in this study scored <20 points (Table 1). However, the HDS-R was developed as a screening test for dementia, with recommendations that it be interpreted in conjunction with additional clinical data [23]. Therefore, we used the N geriatric scale for mental status (NM scale) to evaluate dementia severity. The NM scale is scored by observing an individual’s ADL. The maximum score is 50 points, and the scores are classified as follows: normal (48–50), borderline (43–47), mild dementia (31–42), moderate dementia (17–30), or severe dementia (0–16) [25]. Our clients’ dementia severity, according to the NM scale, was as follows: mild (n = 1), moderate (n = 2), and severe (n = 3); Table 1. Concerning pharmacotherapy, no clients had been prescribed medication to treat dementia, and two clients had been prescribed psychotropic drugs. One client had undergone cognitive rehabilitation. One client regularly and two clients irregularly attended recreation and group exercises held at each facility. No clients received occupational therapy.

Physiotherapy was administered to clients between 2 and 3 times per week on an individual basis within the facilities where the PTs worked. Each treatment session lasted from 15 to 20 minutes; however, the total interaction time, which included the time taken to walk to the physiotherapy room and time to prepare for the session, was 20 to 30 minutes per session. The objectives of the physiotherapy sessions included maintaining and improving the capacity of the older adults for ADL, maintaining and improving the ability to assume sitting and standing positions, and preventing falls. The exercise program included passive stretching, active exercises, active assisted exercise, leg presses, functional activity training, gait training, weight shifting in the sitting position, and ascending and descending stairs. Gait training was performed as assisted walking between the parallel bars, assisted walking with a walker, or independent walking based on the client’s physical function.

### Data collection

Data were collected from January to September 2017. Interviews with the PTs and observations of their physiotherapy sessions and their clients were performed at the facilities where the PTs worked.

With permission from the facilities where the PTs worked, MY observed the physiotherapy sessions of the PTs and their clients while recording the sessions using a video camera. This approach was adopted to enable the authors to become informed concerning the details of the physiotherapy sessions and for the PTs to reflect on the physiotherapy sessions and to speak more freely during the interviews. Upon completion of a single physiotherapy session, MY proceeded to interview the PTs in a room within each PT’s facility.

For the interviews, we used a semi-structured interview guide with the following three open-ended questions: (1) “Please tell me about the client’s physiotherapy program and its background,” (2) “Is there anything you noticed while watching the video footage of the physiotherapy session?” and (3) “Do you have any other thoughts concerning care for older adults with dementia?” PTs were asked to speak freely according to the interview guide questions while watching the recorded video footage of their physiotherapy session with the client(s). MY asked follow-up questions or encouraged the PTs to discuss further details using probes or maintaining a short period of silence [19] to obtain as many details as possible. A single interview session with a PT lasted 70 to 90 minutes. All interviews were audio-recorded.

### Data analysis

Verbatim records in Japanese were generated from audio records of the interviews, and then translated into English after analysis, which was performed by MY, YT, and YY using the M-GTA [16, 17], with a focus on the involvement of PTs with older adults with dementia to encourage engagement in physical activity. The analysis was carried out in accordance with the procedures described in a previous study [15]. First, variations (specific examples) on the analysis theme from verbatim data were extracted, and concepts that could explain other similar examples were generated. New concepts were extracted as the analysis progressed. During this process, other specific examples were sought from the data. Subsequently, categories were formed by examining the relationship between the generated concepts. A story line and results diagram was generated by examining the relationship between categories. Data were not fragmented during analysis. Data were sometimes derived from a single phrase or involved content spanning 1–2 pages. Any ideas that emerged concerning a relationship between two concepts were recorded [16, 17].

This study involved several theoretical and practical considerations. In terms of theoretical considerations, the following steps were performed to enhance credibility [16, 19, 26–28]. Sampling was carried out until data saturation was reached, based on the principles of qualitative research [16, 19]. MY and YY deemed the possibility of saturation because no new concepts were generated when the data from up to the third participant in the snowball sampling were analyzed. No new concepts were generated from the data of the fourth participant either. Data were finally analyzed and supervised by YT and confirmed as saturation. Triangulation [26–28] was performed using measures and observers. Regarding measures, we interviewed PTs with differing years of experience. Data collection consisted of interviews and observations of physiotherapy sessions. Regarding observers, data were analyzed by three researchers and examined repeatedly. For member checking, interviewed PTs confirmed the researchers’ findings and interpretations. In terms of practical considerations, thick descriptions contribute to transferability [28]; therefore, this study provided the following: (i) many of the interviewees’ words in the “Results,” and (ii) information on the characteristics of the PTs and their clients’ ADL, cognitive function, and PT programs in the "Participants and context" section.

### Results

We generated 16 concepts and five categories concerning the involvement of PTs in encouraging participation in physical activity in older adults with dementia (Table 2). The categories are shown in **bold**, the concept names are shown in “double quotations,” and the words of the interviewees are presented in *italics*.

**Table 2:** Categories and concepts.

Category	Concepts
Make structured preparations for clients to begin physical activity	Physiotherapists provide care for people with mobility problems
	Alleviate negative memories of clients
	Understand the client’s condition on the day of physiotherapy
	Maintain familiar movement patterns
Link exercise therapy to a client’s daily life	Prompt a client to perform movements appropriately
	Non-verbal reminders to induce movements
	Specify markers for self-pacing
	Convey information on how to perform movements in an actual living environment
	Provide functional training in a simulated environment
Discover changes in daily life	Identify activities that the client is naturally good at
	Ascertain recent lifestyle habits
	Notice changes in life function
Ascertain cognitive function	Assess what the client remembers
	Ask the client what they wish to do next
Accommodate client differences	Set goals through considering changes in cognitive function
	Set individual standards for evaluating the degree of independence in ADL

ADL, activities of daily living

**Fig 1:**
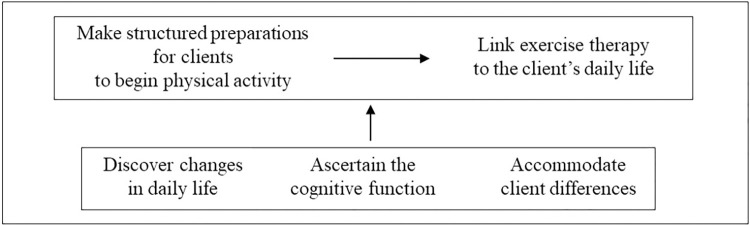
Involvement of the PTs leading to participation in physical activity among older adults with dementia.

The PTs made a concerted effort to **make structured preparations for clients to begin physical activity** to encourage exercise/physical activity. Endeavors to **make structured preparations for clients to begin physical activity** through the **link exercise therapy to a client’s daily life** entailed PTs’ concentration on clients before and during the implementation of the exercise program. Three concepts of interaction, namely, **discover changes in daily life**, **ascertain cognitive function**, and **accommodate client differences**, served to facilitate this process (Fig 1).

### Make structured preparations for clients to begin physical activity

PTs provide care for people with mobility problems. The PTs stated that older adults with dementia who have problems with physical function should be targeted for physiotherapy and follow an active exercise program as far as possible.

*I try to get them to move; move the whole body as much as possible. If a client is truly bedridden, I would resort to more passive exercises, but otherwise, I try to respect and endorse active exercise in people who have the will and the ability to move on their own. (PT-2)*

*There are clients who tend to experience pain even with passive exercise and are reluctant to perform them. Therefore, if a client has a range of motion in the joint that allows changing of clothes, I think it is best to endorse active exercise. They will not perform the exercise without encouragement. In many cases, they just tend to sit and stare blankly. (PT-3)*

The PTs observed the client to “understand the client’s condition on the day of physiotherapy,” upon meeting a client, such as the client’s physical condition, what the client spoke about, the level of arousal, facial expression, and movement. Based on the results, the PTs decided the timing of physiotherapy and the outline of the exercise/movement training content to be performed on that day.

*First, their eyes are sometimes closed; I call them to see if they will open their eyes or not. I check the extent to which the eyes open. Then, whether they maintain their eyes open. I also check to see if the client’s mood is good or bad on the day. Even if they are in a good mood, they can get agitated and angry. (PT-2)*

PTs “alleviate negative memories of clients,” such as pain, discomfort, and anxiety. PTs were careful not to provoke the bad memories of clients.

*The last thing is to ensure that you say goodbye to the clients after they reach their next destination. This is where they differ from healthy older people. Whereas a person without cognitive decline would be able to go to their next destination on their own, clients become anxious when left alone in a place. Therefore, I check by asking “What are you going to do next?” before taking them there and telling them goodbye. (PT-1)*

*The general rule is to avoid anything that the client does not like. In particular, the client clearly knows what she wants to do, so I do not force her to move when she says, “I do not want to move.” (PT-2)*

PTs emphasized the need for clients to “maintain familiar movement patterns” and understood that having an appropriate movement pattern or pace was related to maintaining movement capacity in ADL.

*There are many clients who maintain function through repetitive movements. (PT-3)*

*He is receptive to familiar things but tends to have challenges with accepting new things. In general, I keep things in the same form and use the same tasks, as in a mechanical order, so that it won’t burden the client. (PT-4)*

### Linking exercise therapy to a client’s daily life

This category comprised concepts that showed how the PTs engaged with the clients when implementing exercise and movement training. A PT observed how a client performed exercises and movements and then “prompted a client to perform movements appropriately.”

*I first ask the client to move. I observe how he moves and assist him as needed. (PT-2)*

PTs also provided “non-verbal reminders to induce movements,” such as incorporating group therapy and setting an environment that would encourage the performance of an intended exercise.

*If I want the client to maintain a sitting position as long as possible without resting on the back of the chair, I gather a few people around a table and have the clients engage in tasks. This is not done as a task or occupational therapy, but rather, it is done when I want to train the client’s postural balance. However, the client is not capable of maintaining the posture through verbal instructions alone, so I shift their focus closer to their hands, which shifts their center of gravity forward. (PT-1)*

*If a client is capable of performing more movement, I let them perform ring tossing by positioning the poles a little further away to achieve greater motion in the trunk. If I try to train the client’s trunk control using exercises that require the client to be in a supine position, I do not think they could easily understand the exercise. I think it would be better if muscle strengthening could be achieved during movement. (PT-3)*

PTs observed clients performing exercises and movements and “specified markers for self-pacing.”

*During the knee extension exercise, if I don’t say anything, the client gradually alternates leg movements from side to side. The movement gradually deviates from extension movements and progresses toward stepping. I told the client to “continue” because I wanted him to continue the knee extension exercise on one side. (PT-3)*

PTs “convey information on how to perform movements in an actual living environment” to clients and caregivers. PTs practiced the movements that clients could easily perform with their clients, based on their current physical condition, within their actual living environment. They also educated caregivers on how to perform the movements their clients were familiar with, how to care for them, and how to arrange the environment to facilitate movement.

*There are many people who refuse to exercise as their dementia progresses. Therefore, there are cases for which I assess whether the client can perform some movement using functional activities and observation of movement in the client’s usual environment. If the client tells me, “I am going to the toilet now, so I cannot do the rehabilitation (physiotherapy),” I accompany them by saying, “Alright, then I will go with you.” I also speak with the staff for that floor after assessing the room’s environment and toilet movements and identifying how the client can easily move in that environment. (PT-4)*

PTs “provide functional training in a simulated environment.” This is a common methodology used in physiotherapy. The previously mentioned concept, “convey information on how to perform movements in an actual living environment,” may be the approach that is more frequently used for older adults with dementia.

*There are clients who perform “standing while holding” parallel bars*, *or if capable of doing so on their own*, *use THERABAND and “release one hand off the parallel bar and practice raising of clothing up and down”* as *simulated training*. *(PT-4)*

### Discover changes in daily life

This category comprised three concepts that emerged in relation to the PTs’ discovering past, present, and future ADL of the clients, namely, “identify activities that the client is naturally good at,” “ascertain recent lifestyle habits,” and “notice changes in life function.”

*This client was apparently very active before dementia. He used to love driving cars, and even after he stopped driving, he used to go out by bicycle. He likes moving, and I guess he is enjoying it. He self-propels the wheelchair quite a lot. People around the facility often mention, “Oh, I saw Mr. O there.” (PT-2)*

*In the mornings she asks me, “Who are you, again?” before saying, “Good morning.” She comes to me and asks me, “When is my turn for the rehab (physiotherapy)?” so she recognizes that she is someone that does physiotherapy and that I am her therapist. She is just unable to remember my name. (PT-1)*

*There was a case of a client who had difficulty controlling her toilet needs, feeling uneasy and often saying, “I want to go to the toilet, I want to go!” but calmed down after a balloon was inserted. She had residual urine and difficulties emptying her bladder on her own, which is why a balloon was inserted. I was worried that balloon insertion might make her even more uneasy and that she might pull out the tube, but it was completely the opposite–she became very calm. (PT-4)*

### Ascertain cognitive function

PTs observed the body movements of clients while they performed their routine exercises and movements and “assessed what the client remembers.” Alternatively, they “ask the client what they wish to do next.” The PTs would then ascertain changes in cognitive function based on the client’s movements or responses.

*We have been doing this for a very long time, so the client himself remembers it and does it along with me. At this point, I keep the program unaltered and encourage the client to perform the same exercise to prompt the movements that he remembers. (PT-2)*

*When I ask, “What do you want to do?” those who can answer are those capable of making their own decision, and I have to ask a follow-up question for those clients who do not know what they want to do. For these latter clients, I provide more options for them to answer the question, such as “Toilet? Remove your clothes? Put on your clothes? Cold? and Hot?” Sometimes, a client would just continue to repeat, “No, No.” (PT-1)*

### Accommodate client differences

PTs “set goals through considering changes in cognitive function” and supported the physical activity of clients: “set individual standards for evaluating the degree of independence in ADL.”

*It depends on the client. There are people who, with practice, have become capable of bathing at home independently. It is an issue I consider mostly on a case-by-case basis. (PT-1)*

*He is capable of this much movement now, but at one point, he scored 0 points on the HDS-R (simplified intelligence assessment scale). Sometimes I think that movements are different. There are people who would score high points and still be incapable of going to the toilet, whereas there are those who only score 7 or 8 points but are capable of going to the toilet without assistance. (PT-3)*

## Discussion

In this study, we investigated how PTs interacted with older adults with dementia to encourage exercise and physical activity. Previous studies have reported extensive knowledge and experience acquired through interviews with PTs providing physiotherapy to older adults with dementia [6, 13, 14]. This study revealed further PT experience following their involvement with older adults with dementia when providing individualized exercise therapy.

We found that PTs **make structured preparations for clients to begin physical activity** prior to the next step, to **link exercise therapy to a client’s daily life**, with the goal of encouraging older adults with dementia to voluntarily engage in physical activity during their daily life (Fig 1). Living independently is a general aim, even for older adults with dementia. The step to **make structured preparations for clients to begin physical activity** is a channel through which PTs can engage with older adults with dementia while considering their characteristics, and it is an important approach used to **link exercise therapy to a client’s daily life** and to encourage clients to engage in physical activity. While our goal was to determine how PTs encourage older adults with dementia to participate in physical exercise, this approach and process applied by the PTs was an unexpected discovery.

Clients with normal cognitive functions will come to physiotherapy with the understanding that exercise therapy is needed to improve their physical function and ADL. However, older adults with dementia may not be aware that they need exercise therapy because of problems with cognition and evaluation [1]. In such cases, providing an appropriate context for the client may offer an opportunity for exercise therapy. Furthermore, apathy and anxiety about events increase the risk of diminished willingness to participate in exercise [29]. It has been reported that even if patients with Alzheimer’s disease cannot remember the events that caused their emotions, their emotions can be sustained for a long time [30]. If the client feels uncomfortable during exercise therapy, even if this discomfort is not caused by the PT but relates to an issue such as the client’s physical condition, and negative emotions remain, residual negative emotions can adversely affect their feelings toward subsequent exercise therapy sessions or the PT. Exercise and physical activity have multiple benefits for older adults with dementia [9–11], which are enhanced when **structured preparations are made for clients to begin physical activity.**

Hall et al. reported that memory loss is a common symptom of dementia; however, few PTs suggest specific strategies to overcome it, with PTs using practical approaches to address memory loss [14]. The concepts in **linking exercise therapy to the client’s daily life** involved PTs acting appropriately to vary exercise and movement training to suit each client, similar to that reported by Hall et al. [14].

Conflicting concepts were found in relation to two categories: 1) **make structured preparations for clients to begin physical activity** and 2) **link exercise therapy to a client’s daily life**. One of the concepts identified in the former category was “maintain familiar movement patterns.” Experienced rehabilitation specialists emphasize the importance of consistency and becoming familiar with the treatment environment [14, 31]. Physical activity that “maintains familiar movement patterns,” as reported in this study, concerns ensuring therapeutic environments that provide a client with consistency and familiarity. In addition, maintaining familiar movement patterns require movements that the client can perform actively. Procedural memory, a subtype of implicit memory, includes skill learning, pattern learning, and habit learning, and skill learning tasks are often observed to improve with practice [32]. Procedural memory is considered to be predominantly dependent on the striatum, cerebellum, and other subcortical brain regions, which are considered intact until Alzheimer’s disease becomes more severe [32]. Performing familiar movements during physiotherapy provides an opportunity for PTs to verify that the client’s procedural memory in relation to motor skills is retained. In contrast, “specify markers for self-pacing,” found in the **linking exercise therapy to a client’s daily life** category, involves introducing a physical activity that the client is not accustomed to. This concept can be a disadvantage in terms of enabling a client to become familiar with the therapeutic environment. However, in **making structured preparations for clients to begin physical activity**, PTs do not just provide movement patterns that the client is accustomed to but also engage with the client to help acquire movements in different patterns and paces while the client prepares for exercise therapy; thus, contributing to the improvement in the client’s movement capacity.

We considered that the steps to **discover changes in daily life**, ascertain the cognitive function of clients, and **accommodate client differences** were likely to provide sources of information to facilitate the process in terms of the step to **link exercise therapy to a client’s daily life** via the step to **make structured preparations for clients to begin physical activity** (Fig 1). In circumstances where a client does not have dementia, a PT would speak to the client and evaluate their ADL based on information obtained from their conversation. Even when a client has dementia, a PT would still speak to the client. However, due to cognitive decline, the client may forget details of the conversation or be inaccurate in reproducing the information. To supplement this, a PT would ascertain the client’s lifestyle by obtaining information from caregivers and other healthcare specialists to verify the client’s lifestyle before deciding on how best to approach that client.

Varying degrees of deterioration in clients’ physical and cognitive function may have affected our findings. Potential explanations for this observation are that "ask the client what they wish to do next" and "provide functional training in a simulated environment" could be extracted as variations only for clients with better physical and cognitive abilities. However, since this study was conducted up to the point of generating concepts with M-GTA, this has not yet been validated. It is possible that some concepts (involvement) cannot be provided, depending on the physical and cognitive abilities of older adults with dementia, and further validation is needed.

General principles are set out in the American Psychiatric Association (APA) treatment guidelines in relation to caregivers’ attitudes toward patients with dementia [33]. For example, the guidelines recommend not confronting patients to face their impairment; remaining calm, firm, and supportive; and avoiding unnecessary changes, and the concepts extracted in this study are consistent with these general principles. In addition, according to the 2017 Japanese Clinical Practice Guidelines for Dementia [34], anxiety is an important symptom that can cause or induce behavioral and psychological symptoms of dementia. The first step is to interact with a patient using a reassuring voice and attitude. Evidence indicates that if caregivers practice person-centered care and use appropriate communication, agitation in persons with dementia is likely to improve [34]. In this study, PTs attempted to mitigate behavioral and psychological symptoms of dementia by alleviating the negative memories of the clients and asking the clients what they wanted to do next, which are techniques likely to be useful in practice.

The interviews in this study were conducted prior to the start of the COVID-19 pandemic. We also asked the PTs about their experiences after the COVID-19 pandemic began. They responded that there had been no change in their approach and attitudes toward older adults with dementia. However, the environment is now more focused on infection prevention, and the daily lives of the clients have been affected. For example, the clients and PTs wear masks for infection control; therefore, both of them have found it challenging to communicate with each other through the use of facial expressions and nonverbal communication. Moreover, the PTs needed to observe their clients more carefully than previously. For admitted clients, opportunities for group exercise and outings have been reduced, and the range of activities has been limited due to zoning in the facility. When family visits were also limited, some clients seemed less willing to participate in physiotherapy. In some cases, when online visitation became available, the person regained their motivation. In the infection prevention response to COVID-19, PTs used the same approaches as those presented in this study while accepting that there were changes in their clients as a result of the changes in their environment.

Considering applicability and transferability, the context of the study was described in our methods section, and many statements of the PTs were presented in the results section. Our findings may be useful in understanding and applying physiotherapy in the medical field. However, the study had limitations in that hospitalization involves a drastic change in the environment, and admitted patients are prone to distress associated with medical procedures. Given the significant differences between hospital-based and long-term care, our results may be poorly applicable in some aspects of medical settings. Future studies are required to confirm the applicability of our findings.

## Conclusion

The PTs in our study provided exercise and movement training by applying various degrees of involvement. PTs made structured preparations for clients to begin physical activity to link with exercise therapy. The involvement of PTs in encouraging client physical activity was observed to help guide older adults with dementia to live more independent lives. Our findings may contribute to older adults with dementia continuing to participate in physiotherapy and benefit from exercise.

## Supporting information

S1 Checklist Standards for Reporting Qualitative Research (SRQR). (PDF)

## References

[1] World Health Organization. Dementia. [Cited 2022 Aug 2]. Available from: https://www.who.int/news-room/fact-sheets/detail/dementia

[2] GBD 2019 Dementia Forecasting Collaborators. Estimation of the global prevalence of dementia in 2019 and forecasted prevalence in 2050: an analysis for the Global Burden of Disease Study 2019. Lancet Public Health. 2022;7: e105–e125. doi: 10.1016/S2468-2667(21)00249-8 34998485 PMC8810394

[3] Alzheimer’s Disease International. World Alzheimer report 2015. [Cited 2023 Apr 27]. Available from: https://www.alzint.org/resource/world-alzheimer-report-2015/

[4] World physiotherapy. What is physiotherapy? [Cited 2022 Aug 2]. Available from: https://world.physio/resources/what-is-physiotherapy

[5] McGilton KS, Campitelli MA, Bethell J, Guan J, Vellani S, Krassikova A, et al. Impact of dementia on patterns of home care after inpatient rehabilitation discharge for older adults after hip fractures. Arch Phys Med Rehabil. 2021;102: 1972–1981. doi: 10.1016/j.apmr.2021.06.006 34242626

[6] Foley T, Sheehan C, Jennings AA, O’Sullivan T. A qualitative study of the dementia-care experiences and educational needs of physiotherapists in the Republic of Ireland. Physiotherapy. 2020;107: 267–274. doi: 10.1016/j.physio.2019.08.006 32026828

[7] Hall AJ, Fullam J, Lang IA, Endacott R, Goodwin VA. Community physiotherapy for people with dementia following hip fracture: Fact or fiction? A qualitative study. Dementia (London). 2020;19: 2750–2760. doi: 10.1177/1471301219857027 31219697

[8] Cations M, May N, Crotty M, Low LF, Clemson L, Whitehead C, et al. Health professional perspectives on rehabilitation for people with dementia. Gerontologist. 2020;60: 503–512. doi: 10.1093/geront/gnz007 30759218

[9] Lewis M, Peiris CL, Shields N. Long-term home and community-based exercise programs improve function in community-dwelling older people with cognitive impairment: a systematic review. J Physiother. 2017;63: 23–29. doi: 10.1016/j.jphys.2016.11.005 27993488

[10] Lam FM, Huang MZ, Liao LR, Chung RC, Kwok TC, Pang MY. Physical exercise improves strength, balance, mobility, and endurance in people with cognitive impairment and dementia: a systematic review. J Physiother. 2018;64: 4–15. doi: 10.1016/j.jphys.2017.12.001 29289581

[11] López-Ortiz S, Valenzuela PL, Seisdedos MM, Morales JS, Vega T, Castillo-García A, et al. Exercise interventions in Alzheimer’s disease: A systematic review and meta-analysis of randomized controlled trials. Ageing Res Rev. 2021;72: 101479. doi: 10.1016/j.arr.2021.101479 34601135

[12] Quick SM, Snowdon DA, Lawler K, McGinley JL, Soh SE, Callisaya ML. Physical therapist and physical therapist student knowledge, confidence, attitudes, and beliefs about providing care for people with dementia: a mixed-methods systematic review. Phys Ther. 2022;102: pzac010. doi: 10.1093/ptj/pzac010 35157773 PMC9155993

[13] Fjellman-Wiklund A, Nordin E, Skelton DA, Lundin-Olsson L. Reach the person behind the dementia -physical therapists’ reflections and strategies when composing physical training. PLoS One. 2016;11: e0166686. doi: 10.1371/journal.pone.0166686 27906996 PMC5132255

[14] Hall AJ, Watkins R, Lang IA, Endacott R, Goodwin VA. The experiences of physiotherapists treating people with dementia who fracture their hip. BMC Geriatr. 2017;17: 91. doi: 10.1186/s12877-017-0474-8 28427333 PMC5399424

[15] Yokogawa M, Taniguchi Y, Yoneda Y. Physical therapy processes-interactions Between a physical therapist and an older client with dementia. Physiother Theory Pract. 2021;14: 1–11. doi: 10.1080/09593985.2021.1913776 33849393

[16] Kinoshita Y. Modified grounded theory approach. 1st ed. Tokyo: IGAKU-SHOIN Ltd; 2020 (in Japanese).

[17] The Japanese Society of M-GTA 2013 Q&A on M-GTA. [Cited 2022 Aug 2]. Available from: https://m-gta.jp/en/qa.html

[18] O’Brien BC, Harris IB, Beckman TJ, Reed DA, Cook DA. Standards for reporting qualitative research: a synthesis of recommendations. Acad Med. 2014;89: 1245–1251. doi: 10.1097/ACM.0000000000000388 24979285

[19] Moser A, Korstjens I. Series: practical guidance to qualitative research. Part 3: sampling, data collection and analysis. Eur J Gen Pract. 2018;24: 9–18. doi: 10.1080/13814788.2017.1375091 29199486 PMC5774281

[20] Japanese Physical Therapy Association. Rigaku Ryouhou Hakusyo 2018. 1st ed. Yokohama: Human Press; 2019 (in Japanese).

[21] Granger CV, Greer DS, Liset E, Coulombe J, O’Brien E. Measurement of outcomes of care for stroke patients. Stroke. 1975;6: 34–41. doi: 10.1161/01.str.6.1.34 1111181

[22] Maki Y, Yoshida H, Yamaguchi H. Computerized visuo-spatial memory test as a supplementary screening test for dementia. Psychogeriatrics. 2010;10: 77–82. doi: 10.1111/j.1479-8301.2010.00320.x 20738811

[23] Imai Y, Hasegawa K. The revised Hasegawa’s dementia scale (HDS-R)–evaluation of its usefulness as a screening test for dementia. J Hong Kong Coll of Psychiatr. 1994;4: 20–24.

[24] Katoh S. Development of the revised version of Hasegawa’s dementia scale (HDS-R). Jpn J Geriatr Psychiatr. 1991;11: 1339–1347 (in Japanese).

[25] Nishimura T, Kobayashi T, Hariguchi S, Takeda M, Fukunaga T, Inoue O, et al. Scales for mental state and daily living activities for the elderly: clinical behavioral scale for assessing demented patients. Int Psychogeriatr. 1993;5: 117–134. doi: 10.1017/S1041610293001462 8292766

[26] Law M, MacDermid JC. Evidence-based rehabilitation: A guide to practice. 3rd ed. Thorofare: SLACK Incorporated; 2014.

[27] Lincoln YS, Guba EG. Naturalistic inquiry. London: SAGE; 1985.

[28] Korstjens I, Moser A. Series: practical guidance to qualitative research. Part 4: trustworthiness and publishing. Eur J Gen Pract. 2018;24: 120–124. doi: 10.1080/13814788.2017.1375092 29202616 PMC8816392

[29] Sondell A, Rosendahl E, Sommar JN, Littbrand H, Lundin-Olsson L, Lindelöf N. Motivation to participate in high-intensity functional exercise compared with a social activity in older people with dementia in nursing homes. PLoS One. 2018;13: e0206899. doi: 10.1371/journal.pone.0206899 30427894 PMC6235314

[30] Guzmán-Vélez E, Warren DE, Feinstein JS, Bruss J, Tranel D. Dissociable contributions of amygdala and hippocampus to emotion and memory in patients with Alzheimer’s disease. Hippocampus. 2016;26: 727–738. doi: 10.1002/hipo.22554 26606553

[31] Ries JD. A framework for rehabilitation for older adults living with dementia. Arch Physiother. 2022;12: 9. doi: 10.1186/s40945-022-00134-5 35361283 PMC8970689

[32] de Wit L, Marsiske M, O’Shea D, Kessels RPC, Kurasz AM, DeFeis B, et al. Procedural learning in individuals with amnestic mild cognitive impairment and Alzheimer’s dementia: a systematic review and meta-analysis. Neuropsychol Rev. 2021;31: 103–114. doi: 10.1007/s11065-020-09449-1 32897482 PMC7889687

[33] Rabins PV, Rovner BW, Rummans T, Schneider LS, Tariot PN. Guideline Watch (October 2014): Practice guideline for the treatment of patients with Alzheimer’s disease and other dementias. Focus (Am Psychiatr Publ). 2017;15: 110–128. doi: 10.1176/appi.focus.15106 31997970 PMC6519627

[34] Dementia Clinical Practice Guideline Development Committee. Clinical practice guideline for dementia 2017. Available from: https://www.neurology-jp.org/guidelinem/dementia/documents/guideline2017.pdf

